# A New Isoflavone Glycoside from *Dalbergia vacciniifolia* (Fabaceae)

**DOI:** 10.3797/scipharm.1112-23

**Published:** 2012-02-27

**Authors:** Ester Innocent

**Affiliations:** Institute of Traditional Medicine, Muhimbili University of Health and Allied Sciences, P.O. Box 65001, Dar es Salaam, Tanzania.

**Keywords:** Fabaceae, *Dalbergia vacciniifolia*, Apioglucoside, Isoflavone

## Abstract

5,5′-Dihydroxy-2′,4′-dimethoxy-7-[(6-*O*-β-d-apiofuranosyl-β-d-glucopyranosyl)-oxy]isoflavone (**1**) was isolated as the major constituent of *Dalbergia vacciniifolia* root bark ethanol extract together with the four known compounds 5,7-dihydroxy-2′,4′,5′-trimethoxyisoflavone (**3**), 5,7-dihydroxy-2′,4′-dimethoxy-isoflavone (**4**), 5-hydroxy-2′,4′,7-trimethoxyisoflavone (**5**) and 7-hydroxy-2′,4′,5′-trimethoxyisoflavone (**6**). Identification of compounds was achieved through extensive analysis of 1D and 2D NMR and MS spectroscopy.

## Introduction

**Fig. 1.**
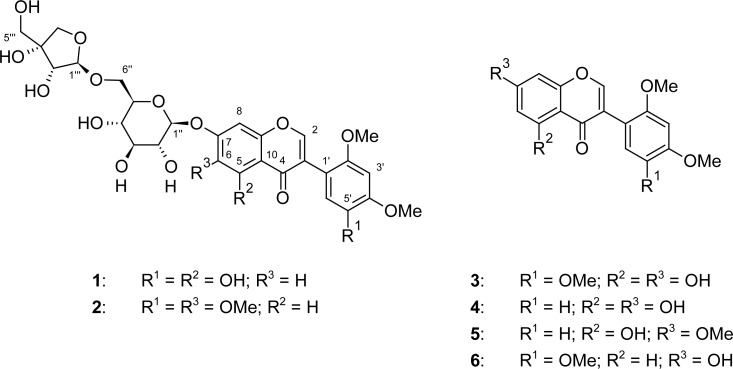
Chemical structure of compounds isolated from *D. vacciniifolia* root barks

*Dalbergia vacciniifolia* Vatke (Fabaceae) is a shrub or small tree of *ca*. 1.3–10 m tall. The plant species grows in coastal bushland and thicket of Tanzania and Kenya where the decoction of the roots is used as a purgative [1, 2]. In the course of our continuing studies on the chemical constituents of this plant species, a new isoflavone glycoside, 5,5′-dihydroxy-2′,4′-dimethoxy-7-[(6-*O*-β-d-apiofuranosyl-β-d-glucopyranosyl)oxy]isoflavone (**1**), from the ethanol extract of the root barks along with other four known compounds 5,7-dihydroxy-2′,4′,5′-trimethoxyisoflavone (**3**) [3–5], 5,7-dihydroxy-2′,4′-dimethoxy-isoflavone (**4**) [3, 6], 5,7-dihydroxy-2′,4′,7-trimethoxyisoflavone (**5**) [7] and 7-hydroxy-2′,4′,5′-trimethoxyisoflavone (**6**) [4] (Fig. 1) was isolated. Recently, another isoflavone glycoside 2′,4′,5′,6-tetramethoxy-7-[(6-*O*-β-d-apiofuranosyl-β-d-glucopyranosyl)oxy]-isoflavone (**2**) was isolated from the stem part of *Dalbergia vacciniifolia* [8]. This is the second report of the occurrence of apioglucoside isoflavones in *D. vacciniifolia.* However, other isoflavone apioglucosides such as biochanin A 7-*O*-[β-d-apiofuranosyl-(1→6)*-*β-d-glucopyranoside] [9], prunetin 4′-*O*-[β-d-apiofuranosyl-(1→6)-β-d-glucopyranoside] [10], 7-methyl-tectorigenin 4′-*O*-[β-d-apiofuranosyl-(1→6)-β-d-glucopyranoside] [11], biochanin A 7-*O*-[β-d-apiofuranosyl-(1→5)-β-d-apiofuranosyl-(1→6)-β-d-glucopyranoside] and tectorigenin 7-*O*-[β-d-apiofuranosyl-(1→6)-β-d-glucopyranoside] [12] have been previously isolated from *Dalbergia* species.

## Results and Discussion

Isolation of compounds from the ethanolic extract of the root barks of *Dalbergia vacciniifolia* by column chromatography on silica gel eluting with 1:4 v/v methanol and dichloromethane yielded compound **1** as a major constituent in the extract. The compound was isolated as amorphous having absorption maxima at 261 and 342 nm. The ESI-MS showed a fragment peak at *m/z* 625 due to [M^+^+H], 647 due to [ M^+^+Na], and *m/z* 659 due to [M^+^+Cl] hence confirming the molecular weight of *m/z* 624 which corresponded to the formula C_28_H_34_O_16_ of compound **1**. Both ^1^H and ^13^C NMR spectra data for compound **1** exhibit characteristic feature of isoflavone skeleton whose ring B is disubstituted. Identification attempts of the aromatic protons in ring B using HSQC, suggested that they were attached to C-6 (δ_H_ 6.46, δ_C_ 100.5) and C-8 (δ_H_ 6.69, δ_C_ 95.5). Furthermore, the ^1^H NMR spectra for these protons showed meta coupling pattern (H-6, *d, J*=2Hz and H-8*, d, J*=2Hz). These chemical shifts and coupling patterns are typical for 5,7 di-*O*- isoflavones [13, 14], because the 6,7-di-*O*-isoflavone carbon signals would resonate at *ca*.δ 104 and 102 for C-5 and C-8, respectively [8, 15]. Presence of the hydroxyl group at C-5 suggests a chelation system with carbonyl group at C-4 (δ, 181.17) due to an intra-molecular hydrogen bonding [8, 13–15]. The 2D H/C correlation using HMBC showed C-6 and C-8 as having many common neighbors such as carbon signals at δ 162.10 (C-6), 163.46 (C-7), 158.01 (C-9), and 109.81 (C-10). In additional, HMBC showed H-8 as having a strong correlation with anomeric carbon of the glucose moiety (δ_C_ 100.42, δ_H_ 4.98) suggesting that the glycosidic linkage was at C-7. The first hydroxyl group in the structure was deduced to be attached to C-5 of the isoflavone because none of the methoxyl protons showed any correlation with carbon signal at δ 162.10 and δ163.46 in the HMBC plot.

The rest of the three aromatic singlets in the ^1^H NMR spectrum were due to aromatic methine protons at δ 8.13 (H-2, *s*), 6.82 (H-6′, *s*) and 6.66 (H-3′, *s*) as established both by H/C correlations such as HSQC and HMBC for compound **1**. The former proton signal is much deshielded due to α-inductive effect of the oxygen and the mesomeric electron withdrawing effect of the β-carbonyl group characteristic for isoflavones [15]. Assignment of the positions of the protons for structure **1**, were unambiguously reached because in the high field region, the ^1^H NMR spectra showed aromatic methoxy proton singlets at δ 3.57 (H-4′) and 3.70 (H-2′), whose corresponding ^13^C NMR signals appeared at δ 57.31 and 56.80, respectively. The HMBC correlation also indicated that these methoxyl groups were attached to C-2′ and C-4′ at δ 152.74 and δ 141.89, respectively, while the signal at δ 148.47 which did not appear in the plot was attributed to the second hydroxyl group in structure **1**. These observations are consistent with other findings which reported the occurrence of isoflavonoids having 2′,4′,5′,7-substitution pattern in *Dalbergia* species [13, 14] and particularly 2′,4′-dimethoxyl substitution in *Dalbergia vacciniifolia* [8].

In the sugar region of the ^13^C NMR spectrum, nine signals were observed which corresponded to two sugar units, one glucopyranosyl and one apiofuranosyl moiety in which three of these signals (δ 74.03, 68.08 and 64.57) were due to methylene (Table 1). The β*-*configuration of the glycosidic linkage was evident from the ^1^H NMR spectrum due to signals at δ 4.98 (*J* = 7.5 Hz) and the observed ^13^C NMR chemical shifts for the anomeric carbons of glucose (δ 100.42, C-1″) and apiose (δ 109.81, C-1′″)[10,11]. The downfield shifts of C-2″ (δ 73.68), C-6″ (64.57) and C-5′″ (δ 68.08) of the sugar moieties suggested an interglycosidic linkage for apiofuranosyl (1′″→6″) glucopyranosyl [10]. Complete assignments of the structures by using both 1D and 2D NMR spectra unambiguously established 5,5′-dihydroxy-2′,4′-dimethoxy-7-[(6-*O*-β-d-apiofuranosyl-β-d-glucopyranosyl)oxy]isoflavone (**1**) as a new compound.

The aglycone for compound **1** was not isolated in this study, but it has been previously reported to occur in *D. parviflora* [16]. However, its methyl derivative 5,7-dihydroxy-2′,4′,5′-trimethoxyisoflavone (**3**) together with known compounds 5,7-dihydroxy-2′,4′-dimethoxy-isoflavone (**4**), 5-hydroxy-2′,4′,7-trimethoxyisoflavone (**5**) and 7-hydroxy-2′,4′,5′-trimethoxy-isoflavone (**6**) were isolated [3–7]. Isolation of isoflavone apioglucoside from *D. vaccinifolia* which seems to have been reported from other *Dalbergia* species provides for a strong chemotaxonomic relationship with great value in plant biochemistry.

## Experimental

### General experimental procedures

CC: silica gel (Merck, 230–400 Mesh, petroleum ether/dichloromethane/methanol); TLC: silica gel (60 F_254_, Merck) precoated on plastic or aluminium plates; visualization: UV/VIS or anisaldehyde reagent [17]; FT-IR: Shimadzu 8400; UV-VIS: 168 diode array detector; 1D and 2D NMR: either Bruker Avance DRX 500 NMR spectrometers, operating at 500 MHz for ^1^H NMR, and 150 MHz for ^13^C NMR (δ= 0; TMS internal standard); MS: ESI mass spectrometer operating at 70 eV.

### Plant materials

*Dalbergia vacciniifolia* root barks (voucher specimen reference No. 1682) were collected from Changanyikeni village in Kinondoni District, Dar es Salaam, Tanzania. The plant specimen was authenticated by Mr. Frank M. Mbago from the Department of Botany, University of Dare s Salaam. The voucher specimen is deposited at the Herbarium at the Institute of Traditional Medicine, Muhimbili University of Health and Allied Sciences

### Extraction and isolation

Air-dried pulverized root barks were soaked sequentially in dichloromethane and then in Ethanol, each two times for 72 h. Repeated column chromatograph of the ethanol extract (17 g) yielded seven fractions; Compounds **3** and **4** were obtained after repeated CC of the 3^rd^ fraction on silica gel eluting with 3:2 v/v ethyl acetate and Petroleum ether while further CC of each of the 2^nd^ and 5^th^ fractions on Sephadex^®^ LH-20 eluting with 1:1 v/v MeOH and CHCl_3_ gave compounds **5** and **6**, respectively. Repeated CC on silica gel eluting with 4:1 v/v CH_2_Cl_2_ and MeOH of the 6^th^ fraction yielded compound **1**.

*5,5′-Dihydroxy-2′,4′-dimethoxy-7-[(6-*O*-β-*d*-apiofuranosyl-β-*d*-glucopyranosyl)oxy]-isoflavone (5-Hydroxy-3-(5-hydroxy-2,4-dimethoxyphenyl)-4-oxo-4*H*-chromen-7-yl 6-*O*-[(2*R*,3*R*,4*R)*-3,4-dihydroxy-4-(hydroxymethyl)tetrahydrofuran-2-yl]-β-*d*-glucopyranoside,* ***1****)*

Brown gum; yield, 695 mg; Anisaldehyde: pink, then black; UV, λ_max_ nm, UV, λ_max_ nm, 262 and 342; IR ν_max_ cm^−1^, 1710, 1025 and 949; MS, *m/z* (% rel. int.) 659 [M^+^+Cl]^+^, 647 [M^+^+Na]^+^, 625 [M^+^+H]^+^, calc. for C_28_H_32_O_16_: 624.16896); ^1^H and ^13^C NMR (see Tables 1).

## Figures and Tables

**Tab. 1.** NMR spectral data (500 MHz) for compound **1** from *Dalbergia vacciniifolia*

**Position**	δ_C_*	δ_H_* (*m*)	**H/H COSY**	**HMBC**
2	156.58, CH	8.13 (*s*)	–	3, 4, 9, 1′
3	121.36, qC	–	–	–
4	181.17, qC	–	–	–
5	162.10, qC	–	–	–
6	100.53, CH	6.46 (*d,* 2 *Hz*)	–	10, 6, 7, 8
7	163.46, qC	-	–	-
8	95.46, CH	6.69 (*d,* 2 *Hz*)	–	1′, 6, 7, 9
9	158.01, qC	–	–	–
10	109.81, qC	–	–	–
1′	110.01, qC	–	–	–
2′	152.74, qC	–	–	2′-OMe
3′	101.53, CH	6.66 (*s*)	–	2′ 4′, 1′
4′	141.89, qC	–	–	4′-OMe
5′	148.47, qC	–	–	–
6′	116.95, CH	6.82 (*s*)	–	3, 1′, 4′, 5′
1″	100.42, CH	4.98 (*d*, *J* = 7.5 Hz)	2″	7
2″	73.68, CH	3.33 (*m*)	1″	1″
3″	76.86, CH	3.45 (*m*)	4″, 5″	2″
4″	70.53, CH	3.18 (*m*)	5″, 3″	5″, 6″
5″	76.82, CH	3.83 (*m*)	3″	2″, 4″
6″	64.57, CH_2_	3.48, 3.57 (*m*)	–	–
1′″	109.81, CH	4.82 (*d*, *J* = 3.5 Hz)	2′″	2′″, 3′″
2′″	76.22, CH	3.62 (*m*)	–	4′″, 5′″
3′″	79.47, qC	–	–	–
4′″	74.03, CH_2_	3.67, 3.85 (*m*)	–	1′″, 3′″
5′″	68.08, CH_2_	3.47, 3.88 (*m*)	–	1′″
2′-OMe	57.32, OCH3	3.57 (*s*)	–	2′
4′-OMe	56.80, OCH3	3.70 (*s*)	–	4′

* Samples were run in DMSO (TMS δ = 0).

## References

[1] Gillett JB, Polhill RM, Verdcourt B (1971). Flora of Tropical East Africa. Leguminosae, parts 3&4. Papilionoideae 1&2.

[2] Kokwaro JO (1976). Medicinal Plants of East Africa.

[3] Alvarez L, Rios MY, Esquivel C, Chavez MI, Delgado G, Aguilar MI, Villarreal ML, Navarro V (1998). Cytotoxic Isoflavans from *Eysenhardtia polystachya*. J Nat Prod.

[4] Pérez Gutiérrez RM, Lagunes MF (2004). [Effect of isoflavones from *Eysenhardtia polystachya* as inhibitors of calcium oxalate crystal aggregation]. Rev Mex Cienc Farm.

[5] Burns DT, Dalgarno BG, Gargan PE, Grimshwaw J (1984). An isoflavone and a coumerestan from *Eysenhardtia polystachya*-Robert Boyle's flourencent acid-base indicator. Phytochemistry.

[6] Solange AS, Chalvoilo GM (1995). Unambiguous ^1^H- and ^13^C- NMR Assignments of Iso avones from. *Virola caducifolia*. J Braz Chem Soc.

[7] Jain AC, Kumar A, Gupta RC (1979). Constitution of luteone and parvisoflavones-A and -B and synthesis of their methyl ethers and related isoflavones. J Chem Soc Perkin Trans.

[8] Innocent E, Magadula JJ, Kihampa C, Heydenreich M (2010). Bioactive Isoflavones from *Dalbergia vacciniifolia* (Fabaceae). Nat Prod Comm.

[9] Rao PS, Asheervadam Y, Khalilullah M, Murti VVS (1989). A revised structure for the isoflavone lanceolarin. Phytochemistry.

[10] Ramesh P, Yuvarajan CR (1995). Coromandelin, a new isoflavone apioglucoside from the leaves of *Dalbergia coromandeliana*. J Nat Prod.

[11] Mathias L, Vieira IJC, Braz-Filho R, Rodrigues-Filho E (1998). A new isoflavone glycoside from *Dalbergia nigra.*. J Nat Prod.

[12] Faraga SF, Ahmeda AS, Terashima K, Takaya Y, Niwa M (2001). Isoflavonoid glycosides from *Dalbergia sissoo*. Phytochemistry.

[13] Agrawal PK (1989). Carbon-13 NMR of Flavonoids.

[14] Dewick PM, Harborne JB (1994). The Flavonoids. Advances in Research since 1986.

[15] Jha HC, Zilliken CF, Breitmaier E (1980). Carbon-13 chemical shift assignments of chromones and isoflavones. Canadian J Chem.

[16] Songsiang U, Wanich S, Pitchuanchom S, Netsopa S, Uanporn K, Yenjai C (2009). Bioactive constituents from the stem of *Dalbergia parviflora*. Fitoterapia.

[17] Stahl E (1969). Thin layer chromatography: A laboratory handbook.

